# Croup

**Published:** 1880-12

**Authors:** 


### CROUP

Is inflammation of the wind-pipe, which causes it to be contracted, making breathing difficult, and sometimes impossible. Croup is the result of cold, though there is generally an hereditary disposition to it.

It comes on with an increased frequency in breathing in the evening ; the next morning the child is better, and at night worse again, and on the third or fourth night, or sooner, it is regular croup. The child is restless, breathes hard, wheezes and has a dry cough. If proper remedies are applied the first or even the second night, but few children will die of croup. Give two teaspoonsful of epsom salts and put the child to bed ; then apply mustard draughts, or "mustard leaves" to its feet. Wring out a flannel cloth in hot water, and wrap it around the neck as warm as it can be borne, protecting the bed with dry cloths. If the breathing is not easier, and the skin not getting moist in 3 or 4 hours, mix half a teaspoonful each of powdered alum and ipecac in half a glass of tepid water and give it. If it does not vomit in ten minutes repeat the dose with a teacup of warm water every five minutes until there is free vomiting. If the bowels are constipated use a "Nelaton Suppository for Children" every 3 hours, until there is a free passage.

If this treatment is applied early, it will seldom fail. If, however, the disease is well established before treatment is commenced, and the above plan of treatment should fail to afford relief in 12 hours, then give ten grains of calomel, mixed with one drachm of saltpetre, called nitrate of potash ; divide into twelve powders and give one every two hours.

*Recapitulation.*—When a child under seven years of age presents symptoms of croup, give two teaspoonsful of epsom salts, put it to bed and apply mustard draughts and cloths wrung out in hot water around the neck. If no decided improvement in three or four hours, give an emetic of half a teaspoonful each of alum and ipecac in half a glass of tepid water, repeating every ten minutes if necessary, until free vomiting is produced.

Every mother should keep on hand, for such an emergency, a bottle of syrup of ipecac, and give two teaspoonsful every ten minutes till free vomiting is induced. This treatment, with good nursing, at the commencement of an attack of croup, will generally be sufficient to effect a cure. During convalescence the little patient should have good nourishment.

